# Comprehensive Complication Index Predicts Cancer-Specific Survival of Patients with Postoperative Complications after Curative Resection of Gastric Cancer

**DOI:** 10.1155/2018/4396018

**Published:** 2018-11-19

**Authors:** Ru-Hong Tu, Jian-Xian Lin, Ping Li, Jian-Wei Xie, Jia-Bin Wang, Jun Lu, Qi-Yue Chen, Long-long Cao, Mi Lin, Chao-Hui Zheng, Chang-Ming Huang

**Affiliations:** ^1^Department of Gastric Surgery, Fujian Medical University Union Hospital, Fuzhou, China; ^2^Department of General Surgery, Fujian Medical University Union Hospital, Fuzhou, China; ^3^Key Laboratory of Ministry of Education of Gastrointestinal Cancer, Fujian Medical University, Fuzhou, China; ^4^Fujian Key Laboratory of Tumor Microbiology, Fujian Medical University, Fuzhou, China

## Abstract

**Objective:**

To investigate the prognostic impact of postoperative complications for patients with gastric cancer.

**Methods:**

Postoperative complications of patients undergoing radical gastrectomy for gastric cancer were reviewed. The severity of complications was graded by the CCI and C-D classification.

**Results:**

A total of 5327 patients were included in the study. Complications were observed in 767 patients. When the C-D classification system was applied, for patients with grade I–II complications, the length of stay (LOS) of those with high CCI (CCI ≥ 26.2) was significantly longer than that of patients with low CCI (CCI < 26.2) (*p* < 0.001). The 5-year cancer-specific survival rate of patients with complications (52%) was lower than that of patients without complications (61%) (*p* < 0.001). Analysis of the factors associated with prognosis in patients with gastric cancer revealed that complications were independent risk factors for specific survival. When CCI was used to classify complication severity, the 5-year cancer-specific survival rate of the high-CCI group was 46.3%, which was lower than that of the low-CCI group (54.9%, *p* = 0.009).

**Conclusion:**

Complication after radical gastrectomy is an independent prognostic factor, and the complication severity as graded by CCI reflects the difference of cancer-specific survival in gastric cancer patients with postoperative complications.

## 1. Introduction

Gastric cancer is one of the major diseases threatening human health. Resection, which is the only possible radical treatment for gastric cancer, has been extensively examined by researchers, and the issue of how to guarantee the efficacy of oncology while maximally improving safety has become a research hotspot. Postoperative complications are commonly reported in patients with gastric cancer in the literature, with an incidence of approximately 4.2%–23.3% [1–7]. Complications not only increase the suffering of patients, prolong LOS, and increase hospitalization costs but also reduce the quality of life (QoL) and can even lead to early death. Studies in recent years have shown that early postoperative complications reflect the short-term postoperative efficacy and may affect the long-term prognosis of patients through a systemic inflammatory response or a suppressed immune system [8–10]. Although many researchers reported that complications may reduce the long-term survival of patients with cancer, few studies have investigated the association between the severity of complications and long-term outcomes. Baba et al. [9] showed that the long-term survival of patients with esophageal cancer was reduced by postoperative complications, and further studies indicated that there was no significant difference in the long-term prognosis of patients with complications of different C-D grades. If it is presumed that the severity of complications is positively correlated with prognosis but no significant correlation between C-D grades and prognosis has been found, this may be explained by the low sensitivity of this index. In recent years, some scholars have proposed adopting CCI in complication severity grading, and the sensitivity of this index is superior to the traditional complication classification indexes [11–13]. Some scholars reported that complication severity graded by CCI can accurately predict the prognosis of patients with colorectal cancer [14]. Therefore, this present study was designed to investigate which complication severity grading system, the CCI or C-D classification, is more applicable for predicting cancer-specific survival in patients with gastric cancer based on the records of complications and prognosis of patients with radical gastrectomy for gastric cancer in this single-center large sample study.

## 2. Materials and Methods

### 2.1. General Information

Patients who were diagnosed with primary gastric cancer and given a radical gastrectomy in Fujian Medical University Union Hospital from January 1996 to December 2014 were retrospectively analyzed. TMN staging was performed according to the UICC staging criteria, seventh edition, 2010 [15].

### 2.2. Treatments

The inclusion criteria were patients with (1) a pathologically definite diagnosis of malignant gastric tumor before surgery; (2) no direct tumor invasion of surrounding organs such as the pancreas, spleen, and liver, no distant metastasis in the liver, lung, or abdominal cavity, and no significantly enlarged lymph nodes around the abdominal aorta according to preoperative chest X-ray, abdominal ultrasound, and abdominal CT; and (3) D1 + *α*, D1 + *β*, or D2 lymph node dissection and R0 resection diagnosed by postoperative pathology. The exclusion criteria were patients with (1) intraoperative peritoneal dissemination or distant metastasis and (2) incomplete pathological diagnosis and follow-up data. According to the second and third editions of the Japanese version of the guidelines for the treatment of gastric cancer [16, 17], the extent of gastric resection was selected, and lymph node dissection was performed. Adjuvant chemotherapy was recommended for patients with advanced gastric cancer or early gastric cancer with lymph node metastasis, and neoadjuvant chemotherapy was recommended for patients with clinical stage III disease by preoperative staging after 2007. Neoadjuvant chemotherapy and adjuvant chemotherapy were defined as at least one cycle of 5-Fu-based chemotherapy.

### 2.3. Postoperative Complications

Postoperative complications were defined as one or more of the following cases occurring postoperatively: postoperative bleeding (anastomosis and abdominal cavity) [18], incision infection [19], anastomotic leak [20], pancreatic fistula [21], duodenal stump fistula [22], chyle leak [23], abdominal infection [19], delayed gastric emptying [24], postoperative ileus [25, 26], postoperative pneumonia [9, 27], cardiovascular complications, liver complications, and urinary complications. The severity of the complications was graded using the C-D classification system [13] and CCI [11, 28], respectively. The CCI was based on the C-D classification. Complications of individual patients were first graded by the C-D classification, and the weighted sum of different grades of complications was calculated, with a final index ranging from 0 (no complications) to 100 (death from complications). A calculation of CCI can be obtained free of charge at http://www.assessurgery.com [11]. According to related studies, a CCI of 26.2 was set as the cut-off point (equivalent to one grade IIIa complication by the C-D classification), and patients with complications were divided into a high-CCI group (group A, CCI ≥ 26.2) and a low-CCI group (group B, CCI < 26.2) accordingly [14].

### 2.4. Prognosis and Follow-Up

Patients were followed up until death or March 2016, with an interval of 3–6 months, using methods such as outpatient service, home visits, mail, and telephone interviews, and the median follow-up time was 35 months. The overall survival time was defined as the time interval between the operation and all-cause death of a patient, and the cancer-specific survival time was defined as the time interval between operation and death due to tumor recurrence and metastasis.

### 2.5. Statistical Processing

Continuous variables were presented as $\bar{x}\pm s$ and analyzed by Student's *t*-test. Categorical variables were analyzed by the *χ* ^2^ test or Fisher's exact test. The survival curve was plotted according to the Kaplan-Meier method, and differences between curves were tested by the log-rank method. Univariate and multivariate analyses of independent prognostic factors were performed by Cox regression analysis, and variables with *p* < 0.10 in the univariate analysis were selected for multivariate analysis. Differences of *p* < 0.05 were considered statistically significant. The SPSS 18.0 statistical package was used for statistical processing (SPSS, Chicago, IL, USA).

## 3. Results

### 3.1. Incidence of Complications

**Figure 1:**
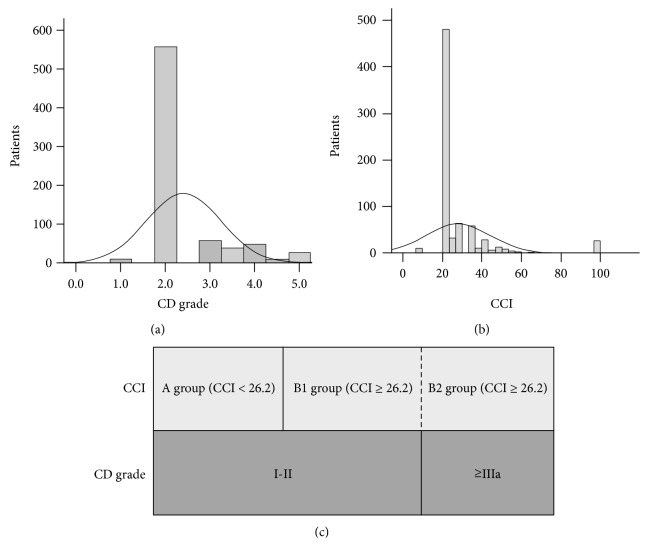
Distribution of complication patients by C-D classification and CCI, respectively. (a) Histogram with probability density curve (solid line) of CD grade in patients with postoperative complications after curative resection of gastric cancer. (b) Histogram with probability density curve (solid line) of CCI in patients with postoperative complications after curative resection of gastric cancer. (c) Horizontal bar graphs show the different division by CD grade and CCI.

A total of 5327 patients undergoing radical gastrectomy for gastric cancer from January 1996 to December 2014 were included in this study, and complications occurred in 767 patients, for an incidence of 14.4%. There were 490 cases (63.9%) with low CCI (CCI < 26.2) and 277 (36.1%) with high CCI (CCI ≥ 26.2) when graded by the CCI system, and there were 582 cases (75.8%) with grade I–II diseases and 185 (24.1%) with grade IIIa–V diseases when graded by the C-D classification system. The distributions of the C-D classification and CCI grading are shown in Figure 1.

General clinical and pathological data of patients with complications are shown in Table 1. Differences in age (*p* < 0.001), ASA (*p* = 0.001), postoperative adjuvant chemotherapy (*p* = 0.004), and operation time (*p* = 0.013) were observed between the low-CCI group and the high-CCI group when graded by the CCI system, and there were differences in age (*p* < 0.001), complications (*p* = 0.004), ASA (*p* < 0.001), and postoperative adjuvant chemotherapy (*p* < 0.001) between grade I–II patients and grade ≥ IIIa patients when graded by the C-D classification system.

### 3.2. Effects of the CCI and C-D Classification Systems on Short-Term Efficacy

The difference between the complication severity grading systems could not be compared directly due to the crossover of patients when graded using these two systems. Grades I–II in the C-D classification included all patients with CCI < 26.2 (group A) and some of the patients with CCI ≥ 26.2 (group B1). Patients with grade ≥ IIIa in the C-D classification were patients with CCI ≥ 26.2 (group B2), as shown in Figure 1(c). Patients in group B1 and group A had the same C-D grades, but not the same CCI scores, and the postoperative LOS of the group B1 was 31.91 ± 18.59 days, which was significantly longer than that of group A (25.1 ± 15.95, *p* < 0.001), and the difference was statistically significant; groups B1 and B2 were different in C-D classification but the same in CCI, and there was no significant difference in LOS between the groups (*p* = 0.717). Thus, CCI could be more reflective of the postoperative LOS for patients with complications (Table 2).

### 3.3. Relationship between Complications and Long-Term Prognosis

The 5-year overall survival rates of patients with complications (*n* = 767) and those without complications (*n* = 4450) were 49% and 60%, respectively, and the difference was statistically significant (log-rank, *p* < 0.001). The 5-year cancer-specific survival rate of patients with complications was 52%, which was significantly lower than that of patients without complications (61%, log-rank, *p* < 0.001) (Supplement Figure 1).

### 3.4. Univariate and Multivariate Analyses of Cancer-Specific Survival in Patients with Gastric Cancer

Factors affecting the survival of patients with gastric cancer after radical gastrectomy were analyzed, as shown in Table 3. The results demonstrated that age (HR = 1.123, *p* < 0.001), neoadjuvant chemotherapy (HR = 1.927, *p* < 0.001), tumor size (HR = 1.104, *p* < 0.001), stage II tumor (HR = 2.765, *p* < 0.001), stage III tumor (HR = 8.759, *p* < 0.001), and complications (HR = 1.194, *p* = 0.006) were independent risk factors for cancer-specific survival.

### 3.5. Relationship between the CCI or C-D Classification and the Long-Term Prognosis of Patients with Complications

**Figure 2:**
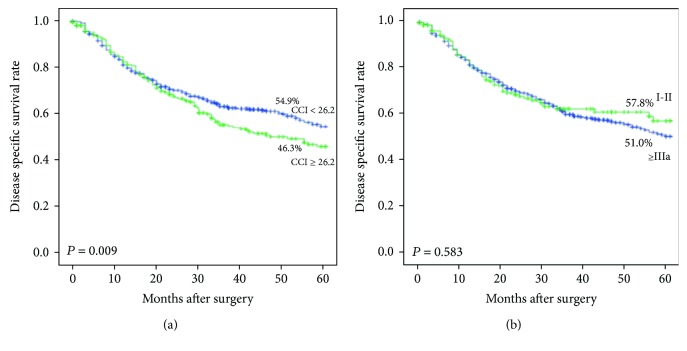
Kaplan-Meier curves of patients with mild and severe postoperative pneumonia according to CCI and CD grade. (a) Disease-specific survival of patients with postoperative complications measured by CCI (*p* = 0.009). (b) Disease-specific survival of patients with postoperative complications measured by C-D classification (*p* = 0.583).

The relationship between CCI, C-D classification, and cancer-specific survival is presented in Figure 2. The 5-year cancer-specific survival rate of the high-CCI group (CCI ≥ 26.2) was 46.3% versus 54.9% in the low-CCI group (CCI < 26.2) (*p* = 0.009). In the C-D classification system, no significant difference was observed in the 5-year cancer-specific survival rate between patients with grade I–II complications and those with grade IIIa complications (57.8% vs. 51.0%, *p* = 0.583).

## 4. Discussion

Complications threaten the lives of patients, prolong the length of the hospital stay, and increase the cost of hospitalization. Surgical teams also spend excessive energy to treat and care for complications. The incidence of postoperative complications of gastric cancer in the literature is approximately 4.2%–23.3%, and the incidence of complications in 5327 patients was 14.4% (767 patients) in the present study, which was similar to previously reported rates. Further studies showed that complications were closely related to the prognosis of patients with gastric cancer.

Reports of the relationship between complications and long-term prognosis after radical gastrectomy for gastric cancer are controversial. Some researchers believe that the complicated postoperative recovery process may inhibit the immune response to the spreading of tumor cells, resulting in a reduced cancer-specific survival rate [9, 10]. Goldfarb et al. [29] further demonstrated that enhancing perioperative cellular immunity while inhibiting excessive catecholamine and prostaglandin responses can effectively reduce the immune suppression of the body and the recurrence and metastasis of tumors. It was also reported that postoperative complications not only prolong the postoperative inflammatory response time but also affect the overall survival and cancer-specific survival of patients after the radical resection of gastric cancer [30]. However, Saito et al. [31] found that there was no significant correlation between complications and the recurrence-free survival rate of gastric cancer and that its predictive value was not as good as that of postoperative changes in CRP. In the present study, to exclude the effect of bias from early complication-induced death and other non-cancer-related death, cancer-specific survival was used as the primary outcome to investigate the relationship between complications and long-term prognosis of gastric cancer. The results showed that postoperative complications are an independent risk factor for cancer-specific survival, and the relationship between the severity of postoperative complications of gastric cancer and the cancer-specific survival rate was reported for the first time in this study. In recent years, CCI has been widely used as an index in clinical trials to evaluate the severity of complications, which is characterized by the adoption of weighted complications that reflect all complications with different severity levels for patients, and its sensitivity is superior to that of traditional complication indexes [11–13]. For example, if patient A has 1 grade II postoperative complication and patient B has 1 grade I postoperative complication and 2 grade II ones, the complications of patients A and B are both graded as grade II using the C-D classification, and CCIs of 20.9 and 28.3 are calculated for patients A and B, respectively, using the CCI classification. In clinical practice, there are great differences between patient A and patient B in terms of complication healing time, the level of systemic inflammation, and immune suppression, and CCI can better reflect the severity of complications in patients A and B. In the present study, a further study revealed that the cancer-specific survival rate of patients with high CCI was lower than those with low CCI, and the difference was statistically significant. When the C-D classification was used to grade the severity of complications, no statistically significant difference in the survival rate was observed between patients with grade I–II complications and those with grade ≥ IIIa complications, which indicated that CCI is more applicable than C-D classification to predict the cancer-specific survival of patients with gastric cancer after surgery. This may be explained by the finding that CCI is more accurate than the C-D classification to reflect the severity of complications and the effects of complications on the level of systemic inflammatory response and the degree of immunosuppression and ultimately affect the prognosis of patients with gastric cancer after the operation.

The correlation between CCI and the cancer-specific survival rate suggests that the active treatment of complications in clinical practice improves the short-term efficacy and reduces the effect of complications on the cancer-specific survival rate. Although the occurrence of some complications is difficult to predict and avoid, clinicians should be vigilant to prevent complications that occur subsequently and take appropriate preventive measures to reduce the number of complications to decrease the CCI. For example, patients with postoperative anastomotic fistula are prone to complicated pulmonary or abdominal infection; long-term bedridden patients are susceptible to developing deep vein thrombosis and pulmonary embolism; patients with the long-term use of broad-spectrum antibiotics are easily complicated with fungal infections or disorders of intestinal flora. Effective measures should be taken to prevent possible secondary complications to reduce the patients' CCIs and improve the long-term efficacy. The present study was a retrospective, single-center, large sample cohort study, and a unified standard was used to assess the severity of complications and the bias induced by the heterogeneity of the objects was reduced to a certain extent. More reliable results, however, still need to be validated in multicenter, large sample prospective clinical trials.

In conclusion, postoperative complication after radical gastrectomy is an independent prognostic factor and the application of CCI in complication severity grading can reflect the difference of cancer-specific survival in gastric cancer patients with postoperative complications.

## Figures and Tables

**Table 1:** Clinicopathological characteristics of patients undergoing radical gastrectomy.

	Total, *N* = 5327	No complication, *n* = 4560	Comprehensive complication index		*p* (CCI < 26.2 vs. CCI ≥ 26.2)	Clavien-Dindo grade		*p* (I–II vs. ≥IIIa)
			CCI < 26.2, *n* = 490	CCI ≥ 26.2, *n* = 277		I–II, *n* = 582	≥IIIa, *n* = 185	
Age ± SD	59.54 ± 11.24	58.98 ± 11.20	61.66 ± 10.72	64.99 ± 10.54	<0.001	62.04 ± 10.85	65.45 ± 10.80	<0.001
Gender					0.458			0.481
Male	4028	3433	376	219		448	147	
Female	1299	1127	114	58		134	38	
Charlson index					0.068			0.004
0–2	5252	4507	480	265		571	174	
≥3	75	53	10	12		11	11	
ASA classification					0.001			<0.001
1–2	5059	4362	458	239		541	156	
≥3	268	198	32	38		41	29	
Neoadjuvant chemotherapy					0.564			0.926
No	5228	4481	476	271		567	180	
Yes	99	79	14	6		15	5	
Tumor diameter ± SD	51.60 ± 27.99	51.09 ± 27.72	55.08 ± 29.41	53.84 ± 29.28	0.576	54.37 ± 28.65	55.47 ± 31.52	0.656
TNM stage					0.924			0.987
I	1226	1068	102	56		120	38	
II	986	863	80	43		94	29	
III	3115	2629	308	178		368	118	
Gastrectomy					0.073			0.188
Open	2719	2326	263	130		306	87	
Laparoscopic	2608	2234	227	147		276	98	
Extent of resection					0.705			0.276
Total	2945	2494	289	162		336	115	
Distal	2249	1958	187	104		229	62	
Proximal	133	108	14	11		17	8	
Operative time ± SD	221.45 ± 71.72	218.93 ± 69.97	236.88 ± 84.93	235.02 ± 86.92	0.778	237.27 ± 85.49	232.93 ± 86.17	0.557
Blood loss ± SD	152.13 ± 274.15	139.86 ± 173.48	188.16 ± 281.02	286.57 ± 875.02	0.077	190.89 ± 272.76	326.75 ± 1055.49	0.092
Adjuvant chemotherapy					0.004			<0.001
No	3226	2655	348	223		413	158	
Yes	2101	1905	142	54		169	27	
Surgical period					0.013			0.317
1996–2005	1473	1299	125	49		137	37	
2006–2014	3854	3261	365	228		445	148	

**Table 2:** Difference in postoperative stay using different measurements in patients with postoperative complications after curative resection of gastric cancer.

	A groups I–II & CCI < 26.2	B1 groups I–II & CCI ≥ 26.2	B2 group ≥ IIIa & CCI ≥ 26.2	*p* ^a^	*p* ^b^
Postoperative stay	25.1 ± 15.95	31.91 ± 18.59	30.8 ± 24.97	<0.001	0.717

*p* ^a^ for A group (I–II & CCI < 26.2) vs. B1 group (I–II & CCI ≥ 26.2). *p* ^b^ for B1 group (I–II & CCI < 26.2) vs. B2 group (I–II & CCI ≥ 26.2).

**Table 3:** Univariate and multivariate analyses for disease-specific survival in patients after curative resection of gastric cancer.

	Univariate analysis		Multivariate analysis	
	HR (95% CI)	*p*	HR (95% CI)	*p*
Age (for 10-year increase)	1.125 (1.076–1.176)	<0.001	1.123 (1.073–1.175)	<0.001
Male sex (vs. female sex)	0.979 (0.878–1.091)	0.699		
Charlson index ≥ 3 (vs. 0–2)	1.126 (0.758–1.670)	0.557		
ASA classification ≥ 3 (vs. 1–2)	1.212 (0.997–1.473)	0.054		
Neoadjuvant chemotherapy (vs. no)	2.088 (1.557–2.799)	<0.001	1.927 (1.436–2.587)	<0.001
Tumor diameter (for 10 mm increase)	1.237 (1.217–1.258)	<0.001	1.104 (1.082–1.126)	<0.001
Tumor stage II (vs. stage I)	3.301 (2.485–4.385)	<0.001	2.765 (2.076–3.683)	<0.001
Tumor stage III (vs. stage I)	12.394 (9.703–15.831)	<0.001	8.759 (6.778–11.318)	<0.001
Open gastrectomy (vs. LG)	1.401 (1.273–1.543)	<0.001		
Total gastrectomy (vs. distal gastrectomy)	1.789 (1.619–1.978)	<0.001		
Proximal gastrectomy (vs. distal gastrectomy)	1.195 (0.857–1.667)	0.293		
Operative time (for 30-minute increase)	1.109 (1.087–1.133)	<0.001		
Blood loss (for 50 mL increase)	1.069 (1.052–1.085)	<0.001		
Adjuvant chemotherapy (vs. no)	1.149 (1.047–1.261)	0.004	0.872 (0.791–0.962)	0.002
Surgical period 2006–2014 (vs. 1996–2005)	0.988 (0.894–1.092)	0.817		
Postoperative complication (vs. no)	1.347 (1.189–1.526)	<0.001	1.194 (1.051–1.357)	0.006

## Data Availability

The data used to support the findings of this study are available from the corresponding author upon request.

## Supplementary Materials

Supplementary Materials Supplement Figure 1: Kaplan-Meier curves of patients with and without postoperative complications: (A) overall survival (*n* = 5327) and (B) disease-specific survival. The 5-year overall and disease-specific survival rates of patients with postoperative complications were significantly poorer than those of patients without postoperative complications (*p* < 0.001 and *p* < 0.001, respectively).
