# Supplementary figures and images for: Comprehensive Complication Index Predicts Cancer-Specific Survival of Patients with Postoperative Complications after Curative Resection of Gastric Cancer
Source: Gastroenterol Res Pract. 2018 Nov 19;2018:4396018. doi: 10.1155/2018/4396018 (PMC6276389; doi:10.1155/2018/4396018)

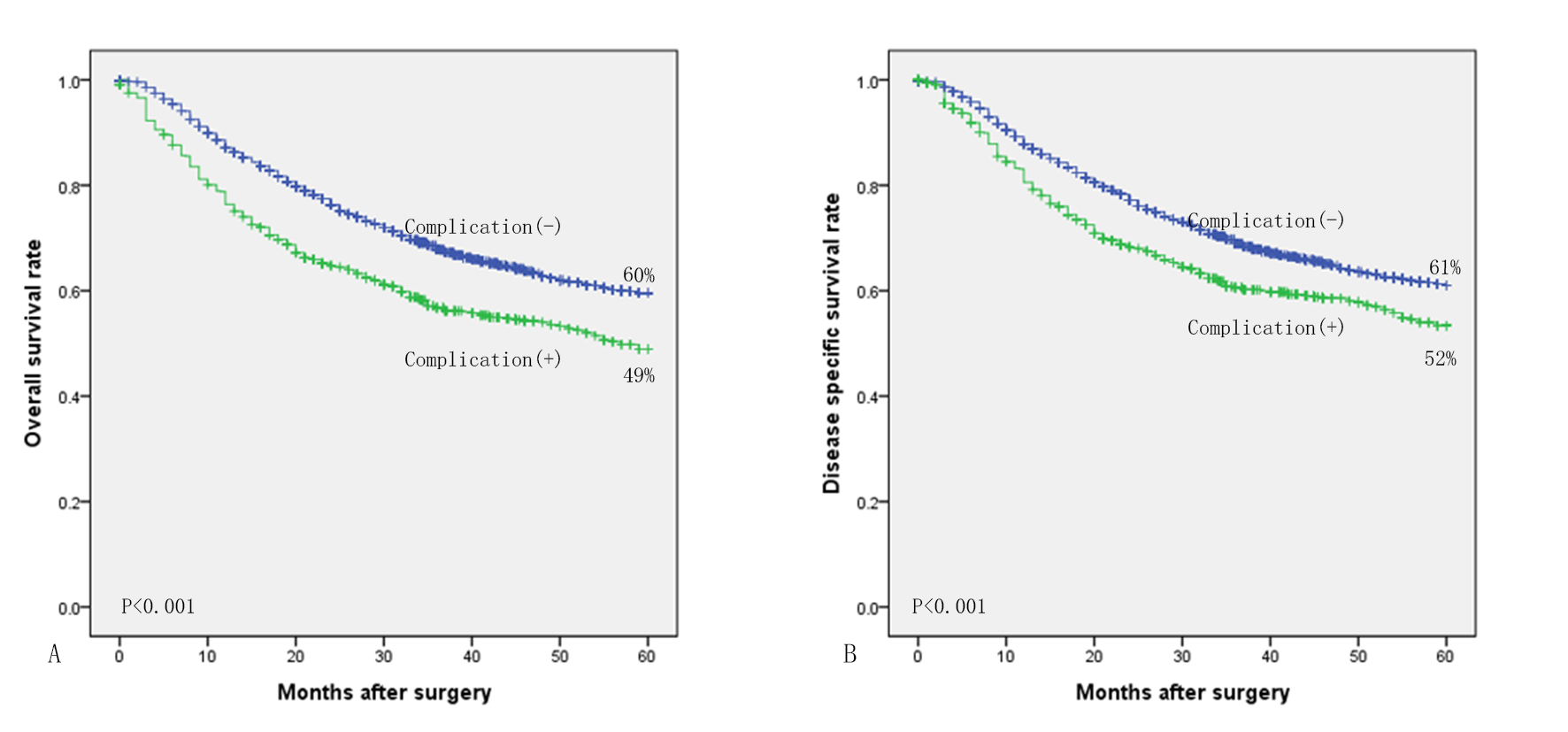

Supplement: Supplementary Materials — Supplement Figure 1: Kaplan-Meier curves of patients with and without postoperative complications: (A) overall survival (n = 5327) and (B) disease-specific survival. The 5-year overall and disease-specific survival rates of patients with postoperative complications were significantly poorer than those of patients without postoperative complications (p < 0.001 and p < 0.001, respectively). [file 4396018.f1.tif]
